# Neutrophils in secondary lymphoid organs

**DOI:** 10.1111/imm.13406

**Published:** 2021-08-30

**Authors:** Laurence S. C. Lok, Menna R. Clatworthy

**Affiliations:** ^1^ Molecular Immunity Unit MRC Laboratory of Molecular Biology University of Cambridge Department of Medicine Cambridge UK; ^2^ Cambridge Institute for Therapeutic Immunology and Infectious Diseases University of Cambridge Cambridge UK; ^3^ Department of Immunology and Cell Biology Graduate School of Medicine Osaka University Osaka Japan; ^4^ Immunology Frontier Research Center Osaka University Osaka Japan; ^5^ Cellular Genetics Wellcome Sanger Institute Hinxton UK

**Keywords:** adaptive immunity, lymph node, neutrophil

## Abstract

Neutrophils are traditionally considered short‐lived, circulating innate immune cells that are rapidly recruited to sites of inflammation in response to infectious and inflammatory stimuli. Neutrophils efficiently internalize, kill or entrap pathogens, but their effector molecules may cause collateral tissue damage. More recently, it has been appreciated that neutrophils can also influence adaptive immunity. Lymph nodes (LNs) are immune cell‐rich secondary lymphoid organs that provide an ideal platform for cellular interaction and the integration of immunological information collected from local tissues. A variety of peripheral stimuli promote neutrophil migration to draining LNs via blood or lymphatics, utilizing differing molecular cues depending on the site of entry. Within LNs, neutrophils interact with other innate and adaptive cells. Crosstalk with subcapsular sinus macrophages contributes to the control of pathogen spread beyond the LN. Neutrophils can influence antigen presentation indirectly by interacting with DCs or directly by expressing major histocompatibility complex (MHC) and costimulatory molecules for antigen presentation. Interactions between neutrophils and adaptive lymphocytes can alter B‐cell antibody responses. Studies have shown conflicting results on whether neutrophils exert stimulatory or inhibitory effects on other LN immune cells, with stimulus‐specific and temporal differences in the outcome of these interactions. Furthermore, neutrophils have also been shown to traffick to LNs in homeostasis, with a potential role in immune surveillance, antigen capture and in shaping early adaptive responses in LNs. Understanding the mechanisms underpinning the effects of neutrophils on LN immune cells and adaptive immunity could facilitate the development of neutrophil‐targeted therapies in inflammatory diseases.

## Graphical abstract

In addition to their role in pathogen defence, neutrophils can also influence adaptive immune responses. Neutrophils can traffick to lymph nodes, at baseline and following inflammatory stimuli, to interact with other lymph node immune cells to shape innate and adaptive immune responses.

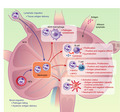

**Abbreviations**
(d)LN: (Draining) lymph node
BM: Bone marrow
CCR: C‐C chemokine receptor
CXCL/R: C‐X‐C chemokine ligand/receptor
DC: Dendritic cell
GVHD: Graft‐versus‐host disease
HEL/CFA: Hen egg lysozyme/complete Freund's adjuvant
HEV: High endothelial venule
IFN: Interferon
Ig: Immunoglobulin
IL: Interleukin
MHC: Major histocompatibility complex
NET: Neutrophil extracellular trap
OVAIC: Ovalbumin immune complex
PMN: Polymorphonuclear leucocyte
PNAd: Peripheral node addressin
PSGL‐1: P‐selectin glycosylated ligand 1
SCS: Subcapsular sinus

## INTRODUCTION

Neutrophils, also known as polymorphonuclear leucocytes (PMNs), are important early effectors of innate immunity. Traditionally considered circulating leucocytes with short half‐lives that are rapidly recruited to inflamed tissues for pathogen killing, neutrophils have also been recognized to more broadly influence innate and adaptive immune responses. One important anatomical location for the latter are lymph nodes (LNs), secondary lymphoid organs where immune cells and antigens congregate, facilitating cellular interactions that generate adaptive immune responses. Here, we will review the evidence for the presence, trafficking and function of neutrophils within secondary lymphoid organs, particularly LNs. Whilst most studies have examined neutrophil behaviour following inflammatory stimuli, we will also discuss emerging data on neutrophil function within LNs under homeostatic conditions.

## NEUTROPHIL LIFE CYCLE

**FIGURE 1:**
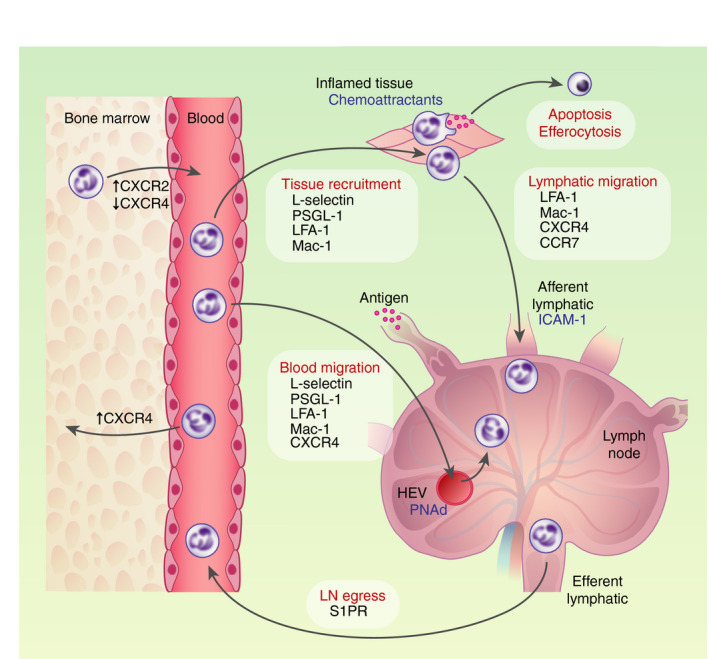
Neutrophil trafficking to LN in homeostasis and following inflammation. Neutrophils are produced in BM and released into blood. Local inflammation results in recruitment of neutrophils to inflamed tissue and to draining LN via blood and lymphatic vessels. Neutrophils also traffick to LN via blood and lymphatic vessels in homeostatic conditions and can egress via efferent lymphatic vessels. Molecules shown to be involved in the mechanisms of neutrophil LN trafficking are listed

Neutrophils are produced in the bone marrow (BM) and released into the intravascular granulocyte pool, consisting of the freely circulating pool in blood, and the marginated pool in liver and spleen, and lungs in mice but not humans [1, 2, 3]. Neutrophils comprise 50–70% of circulating leucocytes in humans and 10–25% in mice [4]. Radiolabelling and adoptive transfer studies showed estimated neutrophil circulatory half‐lives of approximately 10 h in mice and 18 h in humans [5, 6, 7], although estimates were variable in part due to methodological differences [7, 8]. Neutrophil trafficking is regulated by C‐X‐C chemokine ligand–receptor (CXCL‐CXCR) interactions, with neutrophil CXCR4 downregulation mediating neutrophil release from BM, and CXCR4 upregulation on aged murine neutrophils mediating their return to BM for destruction [9] (Figure 1).

## NEUTROPHIL FUNCTIONS

Neutrophils are rapidly recruited to inflammatory tissue sites, and the processes and adhesion molecules involved in this recruitment cascade have been well described [2]. In tissues, neutrophils exert their effector function by mechanisms including phagocytosis, degranulation of cytotoxic proteins, generation of reactive oxygen species and release of neutrophil extracellular traps (NETs). These enable efficient pathogen killing but can also result in collateral tissue damage, as is the case in many chronic inflammatory diseases, for example chronic obstructive pulmonary disease, bronchiectasis and pulmonary fibrosis. Although studies have not specifically examined whether NETosis, degranulation and reactive oxygen species generation occur in LNs, these processes are likely to occur in LNs as well as peripheral tissues. Whilst some neutrophils then undergo apoptosis and efferocytosis by macrophages *in situ*, this accounted for the fate of only one‐fifth of recruited neutrophils in a rat model of immune complex (IC)‐induced glomerulonephritis [10]. In zebrafish, some neutrophils recruited to tailfin injury were observed to migrate away from the injury [11] and exhibited normal phagocytic capacity [12]. Following peripheral inflammation, neutrophils have been shown to traffick to draining LNs (dLNs) via both lymphatic [13, 14, 15] and blood [15, 16, 17] vessels (Figure 1), placing these cells in positions to interact with and influence other LN immune cells.

## LNs: SITES OF IMMUNE CELL INTERACTIONS

Adaptive immunity involves interactions with antigen‐specific cells that are relatively rare populations among circulating leucocytes. Secondary lymphoid organs including spleen, LNs and intestinal Peyer's patches are stations packed with immune cells that concentrate immunological information and facilitate cellular interactions [18]. LNs are present throughout the body, receiving lymphatic drainage from local tissues including skin (e.g. inguinal LN) or mucosa (e.g. mesenteric LN). Each LN has a specific micro‐anatomical arrangement of cells, with circulating B and T lymphocytes entering via peripheral node addressin (PNAd)‐expressing high endothelial venules (HEVs) and guided to their respective locations by stromal cell‐derived chemokines [19]. A variety of innate and stromal cells contribute to the adaptive immune response, such as dendritic cells (DCs) that capture antigen either within LNs (resident DCs) [20] or in peripheral tissues for transport to LNs (migratory DCs) [21] for antigen presentation to T cells, and subcapsular sinus (SCS) macrophages that shuttle antigen draining from lymphatics to follicular B cells [22]. Whilst DCs and macrophages are traditionally considered professional antigen‐presenting cells, other innate cells also play a role. There is now a body of evidence that neutrophils can express major histocompatibility complex (MHC) and costimulatory molecules for antigen presentation, and interact with other LN immune cells to shape adaptive immune responses (see section below).

## NEUTROPHIL TRAFFICKING TO LNs FOLLOWING INFLAMMATION

Neutrophils are capable of transporting antigen to dLNs via lymphatics. Peripheral stimuli such as *Staphylococcus aureus* [13] and Bacillus Calmette–Guérin (BCG) [23] have been shown by microscopy to be captured by neutrophils within murine lymphatic vessels. Following intradermal ovalbumin (OVA) immunization, examination of lymphatic fluid in sheep showed the majority of cells responsible for early (6 h) lymphatic antigen trafficking were neutrophils [24], and similar findings have been observed in mice [25, 26]. However, neutrophil lymphatic trafficking may depend on the nature of the stimulus. Murine studies have shown neutrophil recruitment to dLN following skin challenge with *S*. *aureus* but not with scratch injury [13], thermal injury [27] or herpes simplex virus (HSV) [28]. Four hours following Vaccinia immunization in mice, only 7% of virus‐positive cells in the dLN were neutrophils [29].

Neutrophil entry into dLN via blood vessels has also been shown following murine skin challenges with OVA plus prior immunization to generate OVA‐specific immunoglobulin G (IgG) [15, 25], or with *S*. *aureus* [16, 17]. These murine studies showed that L‐selectin, P‐selectin glycosylated ligand 1 (PSGL‐1) and PNAd were used by neutrophils migrating from HEVs, whereas CXCR4, lymphocyte function‐associated antigen 1 (LFA‐1) and macrophage 1 antigen (Mac‐1) were used by neutrophils migrating from both HEVs and lymphatics [13, 15, 17]. Neutrophil crawling along murine lymphatics was dependent on lymphatic endothelial intercellular adhesion molecule 1 (ICAM‐1) interacting with Mac‐1 [14, 30]. C‐C chemokine receptor 7 (CCR7), required for DC lymphatic migration to LN [21], was required for murine neutrophil lymphatic migration following challenge with complete Freund's adjuvant (CFA) [31] but not *S. aureus* [13, 17] (Figure 1). Examination of human lymphatic endothelial cells from non‐metastatic LNs of cancer patients demonstrated expression of neutrophil chemoattractants by SCS floor and medullary sinus endothelial cells, and expression of CD209 by medullary sinus cells mediating neutrophil adhesion [32].

Simultaneous systemic and local adoptive transfer of differentially labelled, ovalbumin‐IgG immune complex (OVAIC)‐stimulated neutrophils in mice showed that neutrophils entered LNs via both blood and lymphatic vessels [15]. Using laser‐induced sterile injury model, we showed that neutrophil LN recruitment in mice was incompletely inhibited by anti‐PNAd blockade of HEV entry, suggesting lymphatic entry also occurs [33]. However, another study showed anti‐PNAd blockade completely inhibited neutrophil recruitment to LN following murine *S. aureus* infection [17]. Following Influenza vaccination in mice, neutrophil numbers increased more slowly within draining lymphatic vessels than within dLN HEVs [34]. Overall, the precise nature of stimulus‐specific or temporal cues governing different routes of neutrophil entry into LNs remains to be fully determined.

## SURVIVAL OF NEUTROPHILS WITHIN LNs

Whilst the majority of murine neutrophils recruited to dLN following bacterial challenge were shown to be apoptotic [13], some are nevertheless capable of modulating other immune cells [35]. In the circulation in mice, microbiota‐driven neutrophil ageing resulted in an activated phenotype with higher CD11b expression and NET formation [36]. Aged murine neutrophils upregulate CXCR4 [37], which is also involved in neutrophil trafficking into LNs [15], although one study showed downregulation of CXCR4 in murine dLN neutrophils 12 h following Influenza vaccination [34]. Motile BM neutrophils have been shown in recipient peripheral LNs in mice several days following intravenous transfer, although these could represent immature precursors [33]. Neutrophils can migrate to various locations within dLNs, positioning them to interact with cells including SCS macrophages, DCs and lymphocytes.

## NEUTROPHILS AND SCS MACROPHAGES: RESPONSE TO MICROBES

Subcapsular sinus macrophages line the floor of the SCS and capture incoming antigen draining via lymphatics [22, 38]. Pathogens such as *S. aureus* and Influenza virus arriving into the SCS can stimulate murine neutrophil recruitment via HEVs, with neutrophils migrating into the SCS to phagocytose microbes [16, 34]. Neutrophils arriving in dLN formed swarms in two stages, with an initial surge of pioneer neutrophils, followed by a second wave of larger swarms, sufficient to disrupt the SCS macrophage network following *Toxoplasma gondii* infection in mice [34, 39]. Application of an anti‐PNAd antibody in mice blocked neutrophil recruitment and worsened LN and systemic spread of *S*. *aureus* above the effects of clodronate‐mediated macrophage depletion alone, suggesting synergistic effects of neutrophils and SCS macrophages in controlling pathogen spread beyond the dLN [17].

**FIGURE 2:**
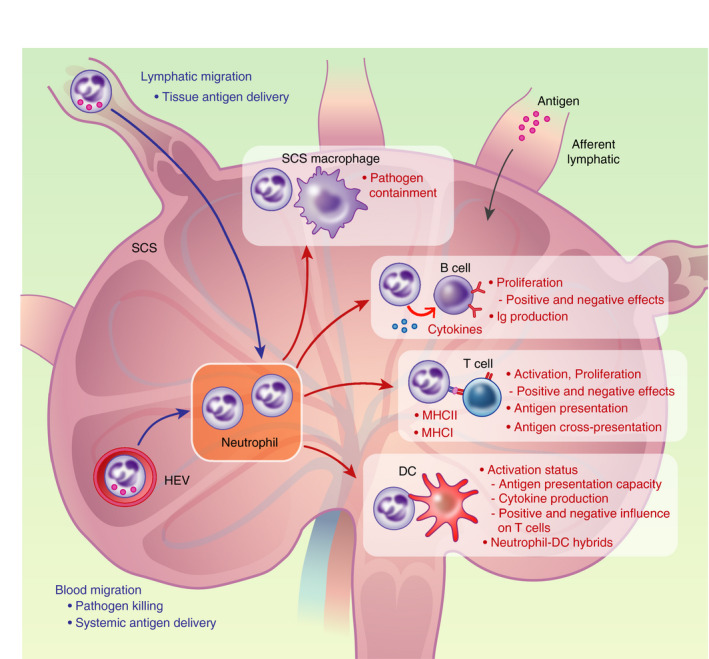
Influence of neutrophils on other LN immune cells. Neutrophils can traffick into LN via blood and lymphatic vessels at baseline and following inflammatory stimuli, delivering peripheral and systemic antigen. Within the LN, neutrophils have been demonstrated to interact with SCS macrophages, DCs, B cells and T cells to influence innate and adaptive responses, with studies showing positive and negative effects

Subcapsular sinus macrophage‐derived interleukin (IL)‐1β has been shown to drive neutrophil LN recruitment in mice following *Pseudomonas aeruginosa* infection [40]. Local infection with Vaccinia virus in mice induced inflammasome activation in SCS macrophages, leading to macrophage pyroptosis and IL‐1R‐dependent recruitment of innate cells including neutrophils [41]. Together, these studies demonstrate that peripheral microbial stimuli lead to neutrophil recruitment to the dLN where they work in concert with SCS macrophages to limit systemic spread of pathogens locally and beyond the dLN (Figure 2).

## NEUTROPHILS AND DCs: INFLUENCE ON ANTIGEN PRESENTATION

Dendritic cells are professional antigen‐presenting cells that capture and process antigen from mucosal and lymphoid tissues, upregulating antigen‐loaded MHCII or MHCI for presentation to CD4^+^ T cells or cross‐presentation to CD8^+^ T cells, respectively, whilst simultaneously increasing the expression of costimulatory molecules such as CD40, CD80 and CD86 required for T‐cell activation. Human neutrophils stimulated with bacteria and fungi activated co‐cultured DCs *in vitro*, leading to upregulation of MHCII and costimulatory molecules on DCs [42], increased IL‐2 secretion and a reduction in IL‐10. Furthermore, *in vitro* murine and human co‐culture studies showed that phagocytosis of antigen‐containing neutrophils by DCs resulted in enhanced antigen presentation and cross‐presentation to CD4^+^ and CD8^+^ T cells [43, 44, 45]. In a house dust mite and lipopolysaccharide (LPS) murine airway inflammation model, neutrophil cytoplasts (enucleated neutrophils formed following NET release) were found in lungs and mediastinal LN, and when co‐cultured with lung DCs led to DC activation promoting IL‐17 and IL‐13 production by CD4^+^ T cells [46]. In contrast, co‐culture of human monocyte‐derived DCs with apoptotic neutrophils resulted in reduced expression of costimulatory molecules on DCs and lower T‐cell stimulation, despite MHCII upregulation [47]. This may reflect species differences, or the differing functionality of *in vitro* versus *in vivo* generated DCs.

Other studies have suggested suppressive effects of neutrophils on DCs in LNs *in vivo*. Following hen egg lysozyme (HEL)/CFA immunization in mice, imaging of dLN *ex vivo* showed only brief neutrophil‐DC contacts of 1–3 min within the first 2 h post‐immunization. At later time points (6–10 h post‐immunization), the number and duration of subsequent DC‐T‐cell contacts were enhanced by neutrophil depletion, despite no further neutrophil‐DC contacts observed, resulting in enhanced CD4^+^ T‐cell response and IL‐2 and interferon gamma (IFNγ) production [48]. *In vivo* studies showed that neutrophils reduced the activation (as measured by MHCII and CD86 expression), antigen uptake and CCR7 expression (and therefore migration) of DCs, via release of myeloperoxidase; neutrophil‐mediated DC suppression consequently reduced dLN CD4^+^ T‐cell activation, including reduced proliferation and IFNγ production, in murine challenge models with *Leishmania major*, delayed skin hypersensitivity and OVA‐induced arthritis [49, 50].

## NEUTROPHILS: CAPACITY FOR DIRECT ANTIGEN PRESENTATION

Neutrophils themselves can show phenotypic features associated with professional antigen‐presenting cells. Whilst human blood neutrophils expressed little MHCII at baseline [51, 52], expression of MHCII and costimulatory molecules including CD80 and CD86 could be induced *in vitro* with inflammatory stimuli such as IgG IC in both murine and human neutrophils [33, 53, 54, 55, 56 ]. Human neutrophils have also been shown to acquire MHCII from macrophages *in vitro* [57]. Neutrophils recruited to dLN following murine *S*. *aureus* challenge upregulated expression of MHCII, CD80 and CD86 [13]. In a murine model of graft‐versus‐host disease (GVHD), neutrophils infiltrated the ileum and migrated to the draining mesenteric LN, where they localized with donor T cells; ileal and mesenteric LN neutrophils showed higher MHCII expression compared to other lymphoid tissues, with a proportion of neutrophils shown to present peptide antigen [58].

There may be stimulus‐specific differences in the induction of neutrophil MHCII expression; we found that murine BM and human blood neutrophils upregulated MHCII expression when stimulated *in vitro* with OVAIC but not with ATP, LPS or *Streptococcus pneumoniae* [33]. However, another study showed that 6 h following peripheral OVA challenge in previously immunized mice, with the potential to generate OVAIC *in vivo*, the majority of OVA‐containing cells in the dLN were MHCII‐negative neutrophils [25]. In Rhesus macaques, 24 h following human immunodeficiency virus (HIV) immunization with adjuvant, some MHCII and CD80 expression was detected on neutrophils within dLN, but at a lower level than monocytes and DCs [59].

Interestingly, some neutrophils have been reported to show a hybrid phenotype. Murine BM granulocytes cultured with granulocyte–macrophage colony‐stimulating factor (GM‐CSF) for 1 week showed large oval nuclei and dendritic morphology, expressing Ly6G (neutrophil marker), CD11c (DC marker), MHCII, CD80 and CD86. These neutrophil‐DC hybrids retained phagocytic and NET capacities in response to bacterial and fungal infections and have been identified in inflamed murine skin and dLN *in vivo* [60, 61, 62, 63]. Similar hybrids have been demonstrated in cultured human CD15^+^ cells [64] and in synovial fluid neutrophils from rheumatoid arthritis patients [65].

Overall, the above studies showed that neutrophils can influence the process of antigen presentation within LNs by interacting with DCs, although activating and suppressive effects have both been demonstrated. Furthermore, neutrophils can upregulate expression of MHCII and costimulatory molecules required for antigen presentation, but the extent to which neutrophils, compared to DCs, contribute to the overall process of antigen presentation within LNs is unclear (Figure 2).

## ADAPTIVE IMMUNITY: NEUTROPHILS AND T CELLS

In Rhesus macaques, neutrophils from dLN 24 h following HIV immunization were able to present antigen to and stimulate proliferation of CD4^+^ T cells *ex vivo*, although not as effectively as monocytes or myeloid DCs [51, 59]. Healthy human neutrophils, when stimulated with GM‐CSF and IFNγ, upregulated MHCII, but studies have not consistently shown antigen‐specific T‐cell proliferation [53, 54]. Neutrophils from Cytomegalovirus‐ and Influenza‐infected human volunteers stimulated antigen‐specific proliferation of autologous CD4^+^ T cells, but less effectively than DCs and monocytes [51]. Murine peritoneal exudative neutrophils presented OVA via MHCII to antigen‐specific CD4^+^ T cells in a contact‐dependent manner, leading to T‐cell proliferation and production of IL‐2 and IL‐17 [66, 67]. OVA‐pulsed murine and human neutrophils upregulated MHCII and increased T‐cell activation in co‐culture [33]; they were also capable of cross‐presentation via MHCI to CD8^+^ T cells, leading to antigen‐specific OTI CD8^+^ T‐cell proliferation and production of IL‐2, tumour necrosis factor alpha (TNFα) and IFNγ, *in vitro* and when pulsed neutrophils were transferred *in vivo* [68, 69]. Murine neutrophil‐DC hybrids could also present and cross‐present antigen to CD4^+^ and CD8^+^ T cells *in vitro* [60, 62].

In mice, neutrophil depletion has been used to assess their importance for T‐cell activation by targeting neutrophil‐expressed antigens (Ly6G and Gr‐1) with depleting monoclonal antibodies. Ly6G is selectively expressed on neutrophils and recognized by the 1A8 clone of anti‐Ly6G antibody, whereas Gr‐1 consists of Ly6G and Ly6C and is recognized by the RB6‐8C5 clone of anti‐Gr‐1 antibody, with neutrophils Gr‐1‐high and monocytes Gr‐1‐intermediate [70]. Neutrophil depletion studies using these reagents have shown conflicting effects on LN T‐cell responses *in vivo* (Table 1). Following murine *S*. *aureus* challenge, anti‐Ly6G neutrophil depletion led to reduced CD4^+^ and CD8^+^ T‐cell numbers [16] and proliferation in the dLN [13]; neutrophils arriving via HEVs interacted with CD4^+^ T cells, but the majority of interactions were transient (64% lasting less than 1 min) and none prolonged [16]. In contrast, DC‐T‐cell interactions in murine LNs occurred in three stages, with the first and third stages consisting of short interactions of several minutes; therefore, neutrophil–T‐cell interactions do not seem to be of a comparable duration [71]. In a GVHD model where neutrophils were recruited from ileum to mesenteric LN as shown using photoconvertible mice, anti‐Ly6G neutrophil depletion resulted in decreased donor CD4^+^ T‐cell proliferation in the mesenteric LN and improved survival [58].

**TABLE 1:** The influence of neutrophils on LN adaptive lymphocytes *in vivo*

Ref.	Peripheral stimulus	Neutrophil depletion	Neutrophil LN entry	Effect on T‐cell response	Effect on B‐cell response
[13]	*S. aureus*	Anti‐Ly6G	Lymphatic	↓CD4^+^ and CD8^+^ proliferation	↓Proliferation
[16]	*S. aureus*	Anti‐Ly6G	HEV	↓CD4^+^ and CD8^+^ cell numbers	↑Plasma cells, ↑IgM, ↑IgG
[26]	OVAIC	Anti‐Gr‐1	Not tested	↓CD4^+^ proliferation	Not tested
[28]	HSV	Anti‐Ly6G	Not tested	No difference CD4^+^ or CD8^+^	Not tested
[48]	HEL/CFA	Anti‐Ly6G Anti‐Gr‐1	Lymphatic	↑CD4^+^ activation (IL‐2, IFNγ)	↑IgG
[58]	GVHD of ileum	Anti‐Ly6G	Lymphatic	↓CD4^+^ proliferation	Not tested

Note

Summary of murine studies using antibody neutrophil depletion to examine the effects of neutrophils on T‐ and B‐cell responses in dLNs *in vivo* following peripheral infectious or inflammatory challenge. Anti‐Ly6G recognizes the neutrophil‐selective Ly6G, whereas anti‐Gr‐1 recognizes both Ly6G and Ly6C.

However, in a murine HSV skin infection model, antigen‐specific CD4^+^ and CD8^+^ T‐cell proliferation in dLN was unaffected by anti‐Ly6G neutrophil depletion [28]. Following HEL / CFA immunization in mice, CD4^+^ T‐cell activation, measured by IL‐2 and IFNγ production, was increased by anti‐Ly6G or anti‐Gr‐1 neutrophil depletion [48]. Peripheral OVA stimulation with prior immunization (resulting in IC generation) in mice led to increased CD4^+^ T‐cell activation and proliferation in the dLN, effects that were abrogated with NIMP‐R14 (anti‐Gr‐1 clone) antibody neutrophil depletion; however, in the same study, neutrophils isolated from OVA‐stimulated dLN also inhibited OTII (OVA‐specific) CD4^+^ T proliferation *in vitro*, in a mechanism involving programmed death ligand 1 (PD‐L1) [26]. In humans, subsets of L‐selectin‐low neutrophils have been identified in blood following LPS challenge and severe injury, and these neutrophils suppressed T‐cell proliferation *ex vivo* in a Mac‐1‐dependent mechanism [72]. Neutrophils with suppressive effects form one of a group of cells termed myeloid‐derived suppressor cells, with mechanisms of suppression including arginase secretion and reactive oxygen species production [73]. Subsets of MHCII^+^ neutrophils have also been identified in healthy murine BM and healthy cattle blood that showed, compared with MHCII‐negative neutrophils, higher oxidative burst capacity but suppressive effects on co‐cultured T cells [74].

A recent *in vitro* study of co‐cultured human blood neutrophils and T cells showed that unstimulated neutrophils had no effect on naïve T‐cell proliferation, suppressed proliferation of early‐activated CD4^+^ and CD8^+^ T cells that showed reduced PD1, TNF and IFN‐γ production and increased proliferation of late‐activated T cells. NETotic but not primed neutrophils suppressed early‐activated T cells, whilst neutrophil contents promoted proliferation of both early‐ and late‐activated T cells [75]. These results may partly explain the conflicting results demonstrated by *in vivo* studies, with the activation status of both neutrophil and T cell affecting the nature and functional outcome of their interaction. In addition, the nature of the stimulus may also determine the overall affect of neutrophils on LN T‐cell responses.

## ADAPTIVE IMMUNITY: NEUTROPHILS AND B CELLS

Neutrophils have been shown to influence B‐cell responses in lymphoid and non‐lymphoid tissues. In human tonsil and small bowel mucosal‐associated lymphoid tissue, neutrophils costained for the B‐cell activating cytokine a proliferation‐inducing ligand (APRIL) [76]. In the human splenic marginal zone (a site for T‐independent B‐cell responses), a subset of ‘B‐helper’ neutrophils have been described that enhanced B‐cell immunoglobulin (Ig) production (IgM, IgA and IgG) in *ex vivo* co‐culture; these neutrophils showed higher MHCII and CD86 expression and produced cytokines involved in B‐cell survival and activation, including B‐cell activating factor (BAFF), APRIL and IL‐21 [77]. These findings, however, were not replicated in an independent study [78].

In contrast to these putative activating effects on B cell, other studies have demonstrated inhibitory effects. In a murine model of lupus, splenic neutrophils preferentially co‐localized with T cells at disease onset, but with B cells in established disease; anti‐Ly6G neutrophil depletion at disease onset, but not in established disease, increased splenic germinal centre B cells and follicular helper T cells, and enhanced autoimmunity [79]. There is also evidence for bidirectional crosstalk between B cells and neutrophils. In pulmonary capillaries, CD18‐dependent neutrophil–B‐cell interactions were observed in mice with a mean interaction time of 4·5 min, with neutrophils acquiring MHCII from B cells; B‐cell deficiency led to reduced apoptosis and increased infiltration of neutrophils in the lungs, resulting in pulmonary fibrosis [80].

*In vivo* murine studies have shown inconsistent results on the effects of neutrophils on LN B‐cell responses (Table 1). Following *S*. *aureus* infection, anti‐Ly6G neutrophil depletion resulted in reduced proliferation of dLN B cells, CD4^+^ T cells and CD8^+^ T cells in one study [13], whilst in another study using the same stimulus anti‐Ly6G depletion led to increased dLN B‐cell numbers and TGFβ‐dependent production of IgM and IgG, but reduced dLN CD4^+^ and CD8^+^ T‐cell numbers [16]. Similar to neutrophil–T‐cell interactions, neutrophil–B‐cell interactions were also mainly transient (54% lasting less than 1 min), but some (17%) interactions were over 30 min in duration [16].

Peripheral HEL/CFA immunization in neutrophil‐depleted mice led to increased B‐cell IgG production and increased CD4^+^ T‐cell activation [48]. In a model of emergency granulopoiesis using conditional neutrophil depletion in lysozyme‐diphtheria toxin mice, following peripheral adjuvant immunization with CFA the early (2 h) wave of neutrophil recruitment (seen in wild type control) was not observed, but the late (7–14 days) wave of neutrophil recruitment was significantly enhanced compared to wild type, with increased neutrophil BAFF production and increases in IL‐17^+^ T cells, B220^+^ B cells and CD138^+^ plasma cells [81]. Overall, *in vitro* and *in vivo* studies have demonstrated both stimulatory and inhibitory effects of neutrophils on T‐ and B‐cell responses in dLN following a variety of peripheral stimuli (Table 1), and further information is needed to understand the mechanisms underpinning these, often opposing, observations.

Therefore, in addition to their role in early pathogen defence, neutrophils are recruited to LNs via blood and lymphatic vessels in response to microbial and inflammatory stimuli and interact with LN immune cells to influence innate and adaptive immune responses in a variety of ways, summarized in Figure 2. Neutrophils with different phenotypes have been described in lymphoid and non‐lymphoid tissues with pro‐ and anti‐inflammatory functions [82], and this heterogeneity has led to recent interests in defining distinct neutrophil subsets with tissue‐specific functions [83].

## NEUTROPHILS IN LNs IN CANCER

Human and murine studies have demonstrated that neutrophils contribute to inflammation in the tumour microenvironment and systemically. In human lung cancer, radiolabelled neutrophils trafficked from blood into tumour tissues *in vivo* [84], and tumour‐associated neutrophils (TANs) comprised up to 25% of isolated tumour cells [85]. Murine TANs have been shown to exhibit both anti‐tumour (N1) and pro‐tumour (N2) phenotypes, with TGF‐β driving a pro‐tumour phenotype [86]; however, the relationship between anti‐tumour TANs and the aforementioned polymorphonuclear‐myeloid derived suppressor cells (PMN‐MDSCs) remain incompletely understood.

Similar to their trafficking to LNs following microbial stimuli, neutrophils can also be recruited to tumour‐draining LNs. In mice, laser‐induced sterile inflammation at skin sites inoculated with colon carcinoma cells led to IL‐17‐dependent neutrophil recruitment to tumour‐draining LNs, with neutrophils entering dLNs via HEVs in a L‐selectin/PNAd‐dependent manner [87]. CD66b^+^ neutrophils were observed in metastatic tumour‐draining LNs in patients with different primary cancers including head and neck, gastrointestinal, thyroid and bladder cancers, with immunohistochemistry showing neutrophils and tumour cells in lymphatic vessels suggesting lymphatic route of entry into LNs [88]. In patients with gastric and oral cancers, higher numbers of dLN neutrophils were associated with more advanced stages of disease and with poorer survival [88, 89]. In patients with early‐stage non‐small cell lung cancer, neutrophil‐DC hybrids have been identified within tumour tissues and draining LNs, and these hybrid TANs stimulated CD4^+^ and CD8^+^ T‐cell responses *ex vivo* [90]. Whilst studies have shown immunosuppressive and therefore pro‐tumour effects of dLN neutrophils [91], and it is traditionally considered that many cancers spread from their primary site via LNs to form distant metastases in other organs, a study of human colorectal cancer samples found distinct phylogenetic origins of LN and distant organ metastases in two‐thirds of patients [92].

## NEUTROPHILS IN LNs IN HOMEOSTASIS

Whilst most studies have investigated the role of LN neutrophils following inflammatory stimuli, there is also evidence that neutrophils are present in tissues at baseline. Parabiosis experiments in mice demonstrated that circulating neutrophils migrated into multiple tissues at baseline with differential functions [93]. Murine tissue neutrophil half‐lives were estimated by mathematical modelling to vary from 9 h in the liver to 18 h in the skin [94], although another study showed that lethal irradiation resulted in murine neutrophils disappearing with half‐lives of 2 days in spleen and 6 days in lungs [6].

Careful examination of murine studies of neutrophil recruitment to dLN following inflammatory stimuli shows the presence of small numbers of neutrophils within control LNs [13, 50]. LNs from healthy aged mice had higher neutrophil numbers with an activated phenotype compared with those from young mice, although aged mice also showed increased systemic inflammation [95]. In sheep, lymphatic fluid draining from the periphery contained neutrophils at baseline, suggesting lymphatic trafficking of neutrophils into LNs [24]. In uninfected Rhesus macaques, CD66^+^ neutrophils were present in inguinal LNs, with higher CCR7 and BAFF expression compared to blood neutrophils [96].

We characterized in detail the presence of neutrophils within different mucosal‐ and skin‐draining LNs in unchallenged mice, showing that neutrophils trafficked at baseline via both blood and lymphatic vessels into LNs where they crawled at a speed of around 6 µm/min [33], comparable to that of LN lymphocytes [20, 97] but faster than that of neutrophils in non‐inflamed skin [98]. LN neutrophils were mainly located in interfollicular T‐cell areas and interacted with DCs, with a mean duration of 5 min, and maximal duration 20 min [33], comparable to neutrophil interaction times with B and T lymphocytes during inflammation [16]. Neutrophils were similarly identified within human LNs from organ donors. Phenotypically, murine and human LN neutrophils showed higher baseline MHCII expression compared to circulating neutrophils [33], with another study also showing similar findings in healthy cattle [74]. *In vivo*, murine neutrophils were capable of delivering systemic IC to peripheral LNs. These results suggested that murine and human neutrophils patrol not only the circulation but also LNs, with a potential role of homeostatic immune surveillance, sampling and delivering circulating antigens to LNs to influence adaptive immunity [33]. A separate study showed that murine neutrophils entered LNs dependent on L‐selectin and, similar to adaptive lymphocytes, egressed via efferent lymphatics back into the circulation dependent on sphingosine‐1‐phosphate receptor (S1PR). Pre‐treatment with L‐selectin to deplete LN neutrophils resulted in blunted neutrophil recruitment to dLN following peripheral *S*. *aureus* infection, suggesting neutrophils play a role in pathogen surveillance locally within LNs [27].

Therefore, in addition to their trafficking to LNs and interactions with other LN immune cells under infectious or inflammatory conditions, neutrophils also traffick to murine and human LNs under homeostatic conditions, with a potential role in antigen surveillance (Figure 2). Whilst human organ donors might have a degree of systemic inflammation, a study of a cohort of organ donors showed that the numbers of neutrophils, monocytes and adaptive lymphocytes in lymphoid and mucosal tissues did not vary significantly with donor clinical status or clinical complications [99]. Human and murine neutrophils exhibit different circulatory dynamics [4, 8], and the immune cellular composition of humans differ from laboratory mice due to differences in microbiological exposures [100]. Therefore, these complementary findings in human LNs [33] are important to confirm the clinical relevance of mouse studies.

## SUMMARY

Whilst neutrophils are traditionally considered circulatory innate effector cells that are rapidly recruited to inflammatory tissues, their diverse roles beyond pathogen killing are increasingly recognized. Following infectious and inflammatory challenges, neutrophils traffick via blood and lymphatic vessels to LNs, where they interact with SCS macrophages, DCs, T cells and B cells to influence innate and adaptive immune responses, for example, by contributing to antigen presentation. However, studies have shown conflicting results on the overall effect of neutrophils on other LN immune cells, and there may be stimulus‐specific and temporal differences in the outcome of these cellular interactions. Furthermore, neutrophils also traffick to LNs at baseline, sampling and delivering antigen to LNs, with a potentially important role in homeostatic immune surveillance. Many chronic inflammatory diseases feature neutrophilic inflammation, yet few neutrophil‐targeted treatments exist. Clarifying the mechanisms underlying the differential contribution to adaptive immune responses may facilitate the future development of therapies that modulate this aspect of neutrophilic inflammation.

## CONFLICT OF INTEREST

None.

## AUTHOR CONTRIBUTIONS

LSCL and MRC co‐wrote the manuscript.
